# Validation aspects of the health of the nation outcome scales

**DOI:** 10.1186/1752-4458-5-20

**Published:** 2011-09-06

**Authors:** Pietro G Lovaglio, Emiliano Monzani

**Affiliations:** 1 CRISP and Department of Quantitative Methods, University Bicocca-Milan, V.le Sarca 202, Milan, Italy; 2 Department of Mental Health, Health Agency of Niguarda, Milan, Italy

## Abstract

**Background:**

The purpose of the current study was the psychometric evaluation of the Health of the Nation Outcome Scales (HoNOS), an instrument developed to meet the necessity of a clinically acceptable outcome scale for routine use in mental illness services.

**Methods:**

The study participants included 2,162 outpatients and residential inpatients (rated on the HoNOS on three occasions during the year 2000) with a range of mental illnesses in different diagnostic groups from ten Mental Health Departments, located in the area of Milan (Italy). Principal Component Analysis, Confirmatory Factor Analysis, Discriminant Analysis and Partial Credit Rasch Model were used to assess two sources of validity: the internal structure and the relationships with other variables.

**Results:**

The results of the 12-item HoNOS demonstrate a significant departure from uni-dimensionality, confirmed by the Rasch analysis (which identified three misfitting items). However, HoNOS scores demonstrate stability and precision of item difficulties over time. Discriminant analysis showed that HoNOS scores have an acceptable level of discriminatory power in predicting the severity of patients' conditions (as represented by setting).

**Conclusions:**

It was concluded that the Italian version of the HoNOS does not measure a single, underlying construct of mental health status. The internal structure validity analysis recommends a note of caution to use a summary index of the HoNOS scores, given the presence of multidimensionality and misfit. Nonetheless, the finding that the instrument is more multidimensional than unidimensional does not preclude the use of the HoNOS as a clinically valid tool for routine outcome assessment. In fact, item scores have demonstrated sufficient reliability (over diagnostic groups and care settings) and high precision in time, indicating that HoNOS items can be utilized as valid measurement instruments in longitudinal analyses.

## Background

In recent years, different standardized assessment tools have been proposed for routine use in the various sectors of mental health. In 1993, the Research Unit of the Royal College of Psychiatrists was entrusted by the UK Government to develop a tool to measure health outcomes in the mental health setting. The Health of the Nation Outcome Scales (HoNOS, [1]) was developed to measure health outcome in response to the UK Government's target to significantly improve the health and the social functioning of the population [2].

Further versions have produced the most recent version, the HoNOS (HoNOS, [3,4]), a rating scale measuring the state of health of adults with mental disorders. After adequate training and supervision, the HoNOS can be quickly and easily filled in by health care workers (nurses, psychiatrists, psychologists and other professionals). It has been officially adopted as an outcome tool in the United Kingdom, Australia, and New Zealand and has been widely used in surveys in numerous European countries.

Many international studies have evaluated the psychometric properties of the HoNOS, in terms of interrater reliability [5-7], usability as a routine tool and validity linked to diagnoses [8], concurrent validity and discriminatory power [7,9,10], construct validity [7,9] and sensitivity to change over time [8,9,11].

Particularly, the necessity of the scale's sensitivity to change over time has been determined by the awareness that a realistic process for the evaluation of mental health services, and in particular the analysis of the effectiveness of the daily practice of community mental health services, must be performed in a longitudinal perspective [12]. Recent examples of longitudinal evaluations (effectiveness of the daily practice of mental health services) of mental health services based on HoNOS are [13-17].

Various authors have presented controversial results regarding the precision of measurement (reliability) and sensitivity to change of the HoNOS: some of whom remained sceptical of its use as routine tool [6,11], while others found it a useful and appropriate instrument for routine administrative data systems [5,8-10,14-17].

Despite the numerous studies suggesting that some of the psychometric properties of the instrument are under-investigated [18] and therefore warrant closer examination, HoNOS is accepted as appropriate for routine monitoring of outcomes for different groups on a range of mental health-related constructs [18-21]. Moreover, HoNOS is gaining increased importance within the development of case-mix systems for the area of mental disorders [22] and also in identifying frequently hospitalized patients with mental disorder [23].

In Italy, the routine use of the HoNOS in psychiatric services remains experimental. Recently, a group of Lombardy Region psychiatrists, perceiving the considerable promise of this instrument, have contributed significantly to the construction of the HoNOS in the Italian context. More specifically, the HoNOS was translated and validated in an extensive and multilayered consensus procedure [24], within the "HoNOS 2 Research" [25], a national study on costs of Mental Health Organization, conducted by the Healthcare directorate of the Lombardy region in 2000. In this study, only interrater reliability was examined [24] The results, based on two professional raters on a small sample of 129 patients, revealed acceptable global interrater reliability (mean weighted k = 0.72; 95% CI = [0.69-0.74]). However, the social subscale presented moderate interrater reliability, particularly for Item 9 (k = 0.60) and Item 12 (k = 0.61).

The aim of the present paper is to assess the psychometric properties of the Italian version of the HoNOS (from here: HoNOS) in meeting the requirements of a clinically acceptable outcome scale. In this regard, psychometric literature has traditionally separated validity into three distinct types: content, criterion, and construct validity. However, contemporary thinking on the subject suggests that these distinctions are arbitrary and that all validity should be conceptualized under one overarching framework, "construct validity". Messick [26] identified five sources of evidence to support construct validity: content, response process, internal structure, relation to other variables, and consequences. These are not considered as different types of validity, but rather, as categories of evidence collected to support the construct validity of inferences made from instrument scores.

In the present paper, we assess selected aspects of construct validity, such as internal structure and relations with other variables in the Italian version of the HoNOS. The "internal structure" examines the extent to which the internal components of a test match the defined construct. Reliability measures (Test-retest, Intraclass correlations, Cronbach's alpha, Split-half) and factor analysis are generally used to obtain evidence of internal structure. To this end, one main aspect of reliability is measurement invariance across time. In fact, a highly desirable characteristic in longitudinal research is that the properties of the instrument used to measure traits do not change from one point in time to another, guaranteeing the accuracy of the traits being measured across different time intervals. Once measurement invariance across time is assured, change in individual growth trajectories is fully attributable to statistical units, rather than the changing psychometric characteristics of the measurement instrument [12].

The category "relations to other variables" is the most extensive category and encompasses many of the traditional, specific validity types: criterion-related validity (including concurrent and predictive validity) as well as much of what was traditionally covered under construct validity (including convergent and discriminant validity). Group-comparison studies (variation in scores across time and in settings, diagnostic groups, etc) are often used in validity research, aimed at testing the hypotheses of expected differences in scores across various groups of examinees, as well as in differential group prediction and relationship studies.

## Methods

### Sample

The analyzed data comes from "HoNOS 2 Research", a study on the costs of Lombardy Region Mental Health Departments (MHD) financed by the National Fund "Programmi Speciali" (ex art. 12; DL 502/92; see [25]) and conducted by the Lombardy Region Health Directorate.

MHDs constitute the main treatment agencies for psychiatric care in the region and express heterogeneous territorial and social realities, including services located in urban and rural areas. In Italy, the standard adopted model of psychiatric care is community-oriented: people affected by mental illness are treated in the community through an integrated network of inpatient and outpatient facilities, located in the catchment area and coordinated by the mental health departments. Outpatient facilities comprise Community Mental Health Centres (CMHC) and Day-Care Facilities, whereas inpatient facilities comprise residential facilities and hospital wards. Patients treated in a community setting are usually hospitalized after a relapse of symptoms occurs.

Patients enrolled in this community-integrated network were identified in one of the following packages of care (setting): the *Community *setting refers to patients treated only in CMHC by psychiatrists or psychologists, with the intervention of other professionals (such as nurses, social workers and rehabilitation therapists); the *Community Day-Care *setting considers patients that, in addition to CMHC activities, were also admitted in Day-Care Centres or in Day Hospitals. The *Residential *setting refers to patients treated in Residential Facilities (intensive care).

During the year 2000, 'HoNOS 2 Research' recruited outpatients (CMHC and Community Day-Care Facilities) and inpatients (Residential facilities) in contact with 10 MHDs located in Milan and the surrounding areas.

As far as study design is concerned, a prevalence cohort of people attending the participating MHDs was selected and a naturalistic three-wave observational design was utilized. All individual who had contact with any MHD setting during an index period of two weeks in January 2000 were eligible for inclusion in the study. Specifically, the study design was intended to routinely deliver the HoNOS to eligible patients on three occasions during the year 2000, through MHD psychologists, psychiatrists and nurses qualified with adequate training regarding distribution criteria and requirements.

Assessments were completed in January 2000 (1^st ^assessment, T1), June 2000 (2^nd ^assessment, T2) and December 2000 (3^rd ^assessment, T3).

Since outcome data were collected as part of routine clinical care and for purposes of quality improvement, no specific written informed consent was obtained.

In order to evaluate the scores' sensitivity over time, of the eligible patients we selected only patients who had complete records (evaluated on three occasions during the year 2000). However, the 'HoNOS 2 Research' was planned to receive information at predetermined moments in time. In situations of change of setting (and new contacts with MHDs), the design was not intended to complete assessments on admission and discharge of patients when particular episodes of care took place. This aspect limits the analysis, particularly for patients who changed settings, as typically occurred for outpatients who, during Community treatment, were admitted to Psychiatric Wards in General Hospitals or Residential facilities. Therefore, the present analysis does not consider patients who changed from a Community-based setting (CMHC or Day-Care settings) to Psychiatric Hospitals or Residential facilities for intensive care during the study period. Thus, only patients remaining in the considered settings are considered as eligible subjects (*Community *setting, the *Community Day-Care *setting and *Residential *setting) for the entire period of the study. The resulting dataset includes 2,162 patients, who constitute 85% of the entire sample of outpatients and Residential inpatients with three HoNOS assessments during 2000 in the 'HoNOS 2 Research'.

The database collecting HoNOS assessments has been merged with the "Psyche" database, the official computerized Regional Information Archive registering social-demographic information, clinical profiles and utilized patient services (type and duration of settings, number and type of contacts, etc.) treated by Lombardy region MHDs.

### Instrument

HoNOS is composed of 12 items (Table 1), related to clinical and psychosocial problems, intended to cover four areas (subscales) of mental health: Behavioural problems (Items 1-3), Impairment (Items 4-5), Symptomatic Problems (Items 6-8) and Social problems (Items 9-12). Each item is scored on a five point scale ranging from 0 to 4. Scores of 2 and over are considered clinically significant, while the total score assesses the severity of patients' mental disorder.

**Table 1:** HoNOS items, subscales and scores

HoNOS Item	HoNOS Total score ans subscales	
Item1 Aggression/overactivity		

Item 2 Self-harm	H1-12 HoNOS total score	

Item 3 Substance abuse	H1-3 Behaviour subscale	

Item 4 Cognitive Impairment	H4-5 Impairment subscale	

Item 5 Physical Impairment	H6-8 Symptom subscale	

Item 6 Hallucinations/delusions	H9-12 Social subscale	

Item 7 Depressed Mood	**Score**	**Description**

Item 8 Other mental/behaviour. problems	0	No problem

Item 9 Relationships	1	Minor problem requiring no action

Item 10 Daily living	2	Mild problem but definitely present

Item 11 Living conditions	3	Moderately severe problem

Item 12 Occupation/activities	4	Severe to very severe problem

### Statistical Analysis: Internal Structure

To examine the internal structure validity of the HoNOS scores, the 12 items were intercorrelated using as the non-parametric Spearman rank method as the measure of association. Furthermore, Principal Component Analysis (PCA), was conducted in order to establish evidence of uni-dimensionality and internal consistency of the scores. Unfortunately, no objective procedures (statistical goodness-of-fit test) for PCA are available to determine exact dimensionality [27]. To this end, Confirmatory Factor Analysis (CFA) appears to be a suitable alternative [27-29]. However, as other measurement experts have noted [29], placing too much reliance on Factor Analysis for validity evidence can result in a very narrow body of empirical support for internal structure validity arguments. Item Response Theory (IRT) techniques are useful to help assess this kind of validity evidence [30], as they can be applied to investigate the consistency of responses to a Guttman pattern and, specifically, if the summative score (total score) from batteries of dichotomous or ordinal items can be used as unidimensional indicators of the "amount" of the underlying latent trait (e.g. mental illness). Among the choice of IRT Models, Rasch models [31] provide a powerful and effective approach for the construction of latent traits (measures) with optimal properties. Among all the IRT models, the Rasch approach alone provides latent measures with *specific objectivity *[31,32], estimating fully separable person parameters (amount of latent trait possessed by persons) and item parameters or difficulties. Item difficulties deal with the "amount" of the underlying latent trait (mental illness) required to respond to each item category.

The Rasch model assumes uni-dimensionality, with all test items measuring the same latent trait. As an alternative approach to assessing dimensionality within the Rasch framework, the use of PCA on original item responses is not employed, utilizing instead Principal Component Analysis of Rasch residuals standardized by their model standard deviation. After the contribution of the latent trait (the Rasch factor) to the data is removed, a Principal Component Analysis of Residuals (PCAR) from a unidimensional data set is expected to extract no principal components [33] and the first eigenvalue v_1 _greater than a proposed fixed cut-point (1.40), signifies a violation of uni-dimensionality.

Nevertheless, the proposed threshold varies with sample size and number of items [27]. Specifically, to fix the criterion eigenvalue thresholds (number of factors to retain), simulation studies [34,35] based on a parallel analysis [36] were performed.

In principle, a number of parallel random data sets are generated via random permutations of the actual data, with the same number of cases and variables as the original dataset (HoNOS Rasch standardized residuals) with the mean eigenvalues (ṽ_i_) of the resulting random data sets used as the comparison eigenvalues.

The accuracy, or reliability, of the measure is provided by the reliability ratio [32] of the latent trait or Person Separation Reliability (PSR), as the proportion of the measure's variance not due to error on total variance. Additionally, the Person Separation Index (PSI), giving standard deviation of the measure in standard deviation units of the errors, reflects the spread of persons along the variable being measured. PSI, ranging from zero to infinity, better highlights the person discrimination because of the ceiling effect of PSR (constrained by an upper boundary).

Furthermore, the Rasch Model furnishes fit statistics, which can be used to evaluate the extent to which the data conforms to the model, thus aiding further decisions concerning the exclusion of certain items in order to improve the measurement properties of the instrument. More specifically, item fit statistics (calculated as the standardized means -across all patients- of the difference between observed scores and those expected by the model on every item) indicate which items are eliciting data with poor fit to the model and why the lack of fit has occurred.

In addition, the use of Differential Item Functioning (DIF) techniques to help detect item bias is also included in this category of types of validity (internal structure) evidence [29,30]. If item difficulties vary across groups (or change over time), after holding mental disturbance at a constant level, the latent trait is then defined differently in each group (time points), making comparisons problematic. If a statistically significant difference in item difficulties by group (or time points) can be demonstrated, then the items are expressed differently for the groups in question. This indicates DIF. DIF effects are computed by a comparison among item difficulties for implied groups (or time points), by means of t-tests, controlling the "experiment-wise" type I error rate and converting the difference in item difficulties for the two groups in a standard t-statistic, by using a pooled standard error [30].

In summary, to examine the internal structure of the HoNOS scores, Rasch fit statistics, PCAR and a range of DIF- based on the Rasch Partial Credit model [32] were employed to establish evidence of uni-dimensionality, internal consistency and the temporal stability of the scores.

### Statistical Analysis: Relations with other Variables

The validity linked to "relationships to other variables" was assessed, firstly, by examination of the variation in HoNOS scores across setting [7,37] and diagnostic groups [18]. Further evidence was provided by Discriminant Analysis in order to predict diagnostic groups and settings of care. To this end, although the HoNOS was not designed to reflect diagnostic differences (e.g. patients with different diagnoses may well be similar on some underlying construct of severity), the total score has proved informative in numerous evaluation studies [see 18 for a review]. Finally, since the present study was restricted to patients who had not changed clinical settings in 12 months, preventing meaningful examination of sensitivity to change of scores over time (responsiveness), the longitudinal analysis was omitted.

## Results

Socio-demographic characteristics and utilized settings for patients at T1 are shown in Table 2.

**Table 2:** Characteristics of the sample at T1 (n = 2162)

Variable	Level	n	%
***Gender***	Female	1152	53.3%

***Education Level***	Illiterate	128	5.9%
	
	Primary School	550	25.4%
	
	Secondary School	916	42.4%
	
	High School/University	501	23.2%
	
	Missing	67	3.1%

***Age Class***	< 25	87	4.0%
	
	25-34	499	23.1%
	
	35-44	554	25.6%
	
	45-54	449	20.8%
	
	55-64	366	16.9%
	
	> 64	204	9.4%
	
	Missing	3	0.2%

***Duration of contact with MHD***	< 1 year	39	1.8%
	
	1-2 years	351	16.2%
	
	3-5 years	390	18.0%
	
	6-15 years	894	41.4%
	
	> 15 years	470	21.7%
	
	Missing	18	0.8%

***Job Status***	Not Employed	452	20.9%
	
	Invalid	455	21.0%
	
	Retired	268	12.4%
	
	Housewife	255	11.8%
	
	Missing	732	34.1%

***Living Situation***	Alone	311	14.4%
	
	With Parents	954	44.1%
	
	With Partner	705	32.6%
	
	With Other Relatives	65	3.0%
	
	Other Situation	31	1.4%
	
	Missing	96	4.5%

***Setting***	Community	1340	62.0%
	
	Community Day-Care	583	27.0%
	
	Residential	172	8.0%
	
	Missing	67	3.0%

### Internal Structure Validity

In order to establish evidence of internal structure validity, we investigated the dimensionality of the scores, their internal consistency and factorial structure at baseline (T1).

To assess the association between ordinal HoNOS items, we used non-parametric Spearman rank correlations. Apart from two correlations exceeding 0.5 (Item 9-Item 10, Item 11-Item 12), low values demonstrated no item duplication and little redundancy among scale items.

PCA was applied to the HoNOS T1-data to explore the dimensionality of the scores (Table 3). Four discrete components (with eigenvalues greater than 1) were identified, accounting for 67% of the total variance. The first component "severity of illness" (λ_1 _= 3.20 accounting 26% of the total variance) encompassed seven of the 12 items including the four items of H9-12 (Social subscale) plus Item 1. ("Aggression/overactivity"), Item 4 ("Cognitive Impairment") and Item 6 ("Hallucinations/delusions"). Then, Item 2 ("Self-harm"), Item 8 ("Other mental and behavioural problems") and Item 7 ("Depressed Mood") were extracted together as a second component (λ_2 _= 1.58, accounting 13% of the total variance), reflecting the close association between these three aspects of mental health.

**Table 3:** Mean of HoNOS Items, consistency analysis and factorial structure (correlations) at T1

HoNOS Item	Mean Score	Alpha if Deleted	Item-total correlation	Factor1	Factor2	Factor3	Factor4
Item 1 Aggression/overactivity	0.53	0.69	0.35	**0.47**	0.28	-0.22	0.25

Item 2 Self-harm	0.09	0.71	0.22	0.23	**0.57**	-0.02	0.12

Item 3 Substance abuse	0.12	0.71	0.16	0.11	0.11	-0.04	**0.78**

Item 4 Cognitive Impairment	0.65	0.68	0.40	**0.53**	-0.30	-0.09	-0.11

Item 5 Physical Impairment	0.37	0.72	0.10	0.22	0.01	**0.50**	0.21

Item 6 Hallucinations/delusions	0.98	0.69	0.38	**0.58**	-0.11	-0.24	-0.12

Item 7 Depressed Mood	0.92	0.73	0.05	0.06	**0.75**	0.25	-0.27

Item 8 Other mental/behaviour. problems	1.34	0.71	0.32	0.16	**0.67**	-0.11	-0.19

Item 9 Relationships	1.74	0.65	0.57	**0.72**	-0.03	-0.15	-0.27

Item 10 Daily living	1.30	0.64	0.63	**0.81**	-0.18	-0.03	-0.15

Item 11 Living conditions	0.89	0.66	0.51	**0.71**	-0.10	0.35	0.06

Item 12 Occupation/activities	0.80	0.67	0.47	**0.63**	-0.09	0.16	-0.03

Item 5 and Item 3 were individually extracted as a third and fourth components, expressing the particular conceptual domains they reflect (physical problems and substance abuse, respectively).

To substantiate these results, the factor structure underlying the data was explored and optimum model fit evaluated using Confirmatory Factor Analysis. Different versions of a single factor model, allowing error variances to correlate, were assessed and evaluated with the Structural Equation Modelling fit indices and particularly the Expected Cross-Validation Index (ECVI), a measure typically used to indicate the best model fit [28].

The smallest value for the ECVI is associated to a one-factor model with error variances' correlations between following couples: Item 11 and 12, Item 7 and 8, Item 7 and 2, Item 8 and 1 (ECVI = 0.22; 90%CI: 0.20-0.26). However, the overall goodness-of-fit Chi-square (X^2^(50) = 416.2 p < 0.001) was highly significant. Further, the Incremental Fit Index (IFI = 0.91), the Comparative Fit Index (CFI = 0.92) and the Root-Mean-Square Error of Approximation (RMSEA = 0.10 with 90%CI: 0.11-0.14) do not reach the fit criteria (values greater than 0.95 are considered as acceptable model fit for the CFI and IFI; RMSEA values below 0.08 are considered as reflecting acceptable fit to the model, with values smaller than 0.05 considered as good fit), signifying that the one factor model does not fit the data adequately.

To examine the weight of item scoring, the percentage contributions of each item (expected to contribute approximately one twelfth to the total score) to the overall mean HoNOS (total score) were calculated. It was found that Item 8 ("Other mental and behavioural problems"), Item 9 ("Relationships"), and Item 10 ("Daily living"), scored high relative to the other items, contributing 14.0%, 18.8%, and 14.3%, respectively, to the HoNOS. Subscales contributed 8.1% (Behaviour), 16.9% (Impairment), 35.8% (Symptom) and 39.2% (Social), respectively (percentages are calculated as the ratio between the means of the subscales and the mean of the HoNOS total score, in order to eliminate the effect of the differing numbers of items per sub scale).

In contrast, low contributions of Item 2 ("Self-harm") and Item 3 ("Substance abuse") to the mean HoNOS (0.7% and 1.3%, respectively) showed that few patients were compromised in these domains.

The apparent multidimensionality notwithstanding, the internal consistency of the HoNOS scores was acceptable (Cronbach's alpha = 0.71, improving with the elimination of Item 5 and Item 7). However, low Item-total correlations for Item 2, Item 3, Item 5 and Item 7 (0.22, 0.16, 0.10, 0.05, respectively) indicated the minimal contribution of these items to global internal consistency.

### Rasch Analysis

In the preliminary results of the (Partial Credit) Rasch Model Item 2 and Item 3 presented disordered categories, meaning that the individual probability of achieving any of the allowable scores, from "no problem" to "severe to very severe problem", does not monotonically increase as the person's mental disorder level increases.

This suggests that the scoring process of these items does not conform to the uni-dimensional latent continuum (mental illness) supposed by the Rasch model.

Hence we merged disordered categories for these items, which revealed no further disordering on a subsequent re-analysis. Moreover, for Item 2 and Item 3, the estimated thresholds ("amount" of the estimated mental illness required to respond to each item category) resulted as not being calibrated along the patients' measure of mental illness. Particularly, none of the patients were compromised enough in these domains to surpass the response's category "no problem".

As far as data dimensionality is concerned, applying a PCAR, the first and the second eigenvalues (v_1 _= 2.25 and v_2 _= 1.56) were greater than the fixed cut-point (1.40) proposed in literature, signifying a violation of uni-dimensionality [38,39].

This was confirmed by the simulations conducted. Since the first and the second eigenvalues of empirical components (v_1 _= 2.25 and v_2 _= 1.56) were greater than their simulated equivalents (ṽ_1 _= 1.66 and ṽ_2 _= 1.48, obtained with 100 random permutations), two additional components underlying standardized residuals were determined. Therefore, both the strength of empirical eigenvalues (as compared with established thresholds or simulated equivalents) suggested a serious departure from uni-dimensionality.

This picture was confirmed by analysis of Rasch Item fit statistics, identifying three misfitting items (unexpected residuals): Item 5 ("Physical Impairment"), Item 7 ("Depressed Mood") and Item 8 ("Other mental and behavioural problems"). For these items, patients with low levels of mental disorder tended to score higher than expected, whereas patients with high levels of mental severity scored lower than expected. With regard to the social subscale items, the less mentally disordered patients tended to score lower than expected, whereas patients with high levels of mental severity scored higher than expected on those items, being particularly evident for Item 10 ("Daily living") and Item 9 ("Relationships").

However, it should be noted however, that these drawbacks occur under the assumptions of the Rasch model (e.g. uni-dimensionality). Further, they may also highlight the scarce evidence of "Response process" validity, defined as evidence of data integrity, meaning that all sources of error associated with the test administration are controlled or eliminated to the maximum extent possible [26].

The violation of uni-dimensionality notwithstanding, HoNOS scores exhibited sufficient evidence of the scores' reliability: the estimated Person Reliabilities in each point in time were 0.746, 0.732 and 0.748, respectively.

To assess temporal stability (precision of the items' difficulties over time) in a Rasch based approach, DIF analysis compared item difficulties across time occasions. To this end, apart from Item 7 (and to a lesser extent, Item 5), the remaining items did not exhibit significant DIF, meaning that items maintain the same level of difficulty between T1 and T3, with item difficulties highly correlating over time: from 0.97 (T2 and T3) to 0.99 (T1 and T3). Finally, item difficulties (for all items) did not show significant differences among diagnostic groups (i.e. items function in the same manner across different diagnoses), whereas groups based on 'settings' have highlighted a significant DIF only for Item 2, suggesting that patients in Residential facilities are over-scored than patients in other settings on this domain.

### Relations with other variables

According to the appropriateness criterion, the more severe the illness, the higher the HoNOS total score. For our aims, the setting is the only external information linked to the patent's severity of mental illness: the Community setting involves patients with low severity, the Community Day-Care setting involves patients with intermediate severity levels, whereas the Residential setting is assigned to patients with higher severity levels.

Table 4 shows that mean HoNOS scores at T1 decreased (whereas variability, measured by the coefficient of variation CV, increased) as the level of illness severity anticipated within these settings decreased. The highest mean total was obtained for Residential/Hospital care, followed by Community Day-Care, and Community setting. The same pattern of a stepwise decrease occurred for seven of the 12 items, the exceptions being Item 3 (and the subscale H1-3 Behaviour), Item 5, Item 7, Item 8 and Item 9. At the item level, *Residential *patients scored higher particularly on Item 10 (Problems with activities of daily living) and Item 11 ("Living conditions"). Mean scores at baseline for implied settings indicate that the scale has some capacity to discriminate between different settings even if the scale seems to confuse, to a certain extent, patients in Residential and Community Day-Care settings.

**Table 4:** Mean (and CV) of HoNOS items by settings at T1 (n = 2095)

HoNOS Items	Care Setting		
	
	*Community*	*Community - Day-Care*	*Residential*
Item 1 Aggression/overactivity	0.38 (150.73)	0.83 (108.64)	0.97 (117.9)

Item 2 Self-harm	0.05 (377.89)	0.15 (241.61)	0.21 (287.8)

Item 3 Substance abuse	0.10 (297.06)	0.19 (208.09)	0.13 (263.4)

Item 4 Cognitive Impairment	0.55 (158.11)	0.63 (138.08)	1.09 (104.4)

Item 5 Physical Impairment	0.33 (172.96)	0.33 (177.37)	0.57 (122.4)

Item 6 Hallucinations/delusions	0.73 (136.64)	1.34 (95.46)	1.51 (82.0)

Item 7 Depressed Mood	0.84 (96.87)	0.98 (98.78)	0.78 (119.0)

Item 8 Other mental/behaviour. problems	1.19 (83.68)	1.53 (77.62)	1.50 (80.0)

Item 9 Relationships	1.57 (66.83)	1.98 (51.98)	1.92 (54.5)

Item 10 Daily living	1.06 (98.06)	1.58 (66.8)	2.04 (50.0)

Item 11 Living conditions	0.69 (125.74)	1.06 (99.13)	1.46 (80.6)

Item 12 Occupation/activities	0.60 (139.72)	0.91 (120.38)	1.09 (105.2)

H1-3 Behaviour total	0.54 (140.77)	1.17 (101.52)	0.93 (112.1)

H4-5 Impairment total	0.88 (124.24)	0.95 (112.63)	1.66 (79.2)

H6-8 Symptom total	2.76 (61.98)	3.84 (55.5)	3.79 (61.3)

H9-12 Social total	3.91 (76.69)	5.52 (60.11)	6.51 (46.1)

H1-12 HoNOS	8.11 (56.41)	11.52 (48.47)	12.90 (41.7)

Further evidence of this type of validity was provided by Linear Discriminant Analysis, conducted to assess whether HoNOS item scores discriminated among different patients' setting (at T1). Firstly, in the stepwise procedure that selected predictors useful to discriminate HoNOS averages among three groups, all HoNOS items were found to be significant (Wilks' Lambda tests), thus demonstrating discriminatory power. Secondly, the utilization of estimated discriminant functions has correctly predicted 77% (leave-one-out cross-validated error rate = 23%), of the involved patients, thus demonstrating that HoNOS scores have an acceptable level of discriminatory power (Table 5), especially for Community and Residential patients, proving less effective for predicting Community Day-Care patients (error rate = 66%).

**Table 5:** Discriminant analysis: predicted settings (% of rows) and error rates based on HoNOS profiles, at T1 (n = 2095)

	Predicted Setting				
**Actual Setting**	***Community Day-Care***	***Community***	***Residential***	**Total** **(Row %)**	**Cross Valid**. **Error rate**

*Community* *Day-Care*	195 (34%)	25 (4%)	363 (62%)	583 (100%)	66%

*Community*	35 (3%)	1278 (95%)	27 (2%)	1340 (100%)	5%

*Residential*	25 (15%)	12 (7%)	135 (78%)	172 (100%)	22%

**Total**	255	1315	525	2095	23%

Furthermore, we examined whether the pattern of scores for each main diagnostic group would reflect the expected prevalence of symptoms. For example, whether patients with schizophrenia and other non-affective psychoses would be expected to have significantly higher scores for psychotic symptoms while those with depression would have higher ratings for the items on 'Depressed Mood.

Table 6 shows the mean scores by main diagnostic groups for HoNOS at T1. The highest mean total score was obtained for the mental retardation and schizophrenic groups, reflecting wide dysfunction across the Impairment and especially the Social Functioning domains (particularly for mental retardation group), but not across the Symptom domain, whose mean total score was higher for depressed patients and with Physiological/behavioural syndromes. Patients with schizophrenia and other non-affective psychoses had significantly higher scores for 'Delusions/Hallucinations'. Patients in the depressed group had highest ratings for 'Depressed Mood'. As might be expected, patients with behavioural syndromes, physical factors and neuroses had the highest scores for 'Other problems'. Patients with mental retardation had higher overall scores than patients with psychotic disorders and personality disorders, with particularly high scores for 'Cognitive Impairment' problems and 'Daily living'.

**Table 6:** Mean of HoNOS Items, by diagnosis at T1 (n = 2079)

	Diagnostic Groups					
**Item HoNOS and subscale**	**Mental retar- dation** **n = 54**	**Schizo-phrenia, schizotypal/delusional** **n = 1192**	**Adult personality/behaviour** **n = 240**	**Depression** **n = 347**	**Physio-logical/Physical behavioural** **n = 19**	**Neurotic, stress-related and somatoform** **n = 227**

Item1 Aggression/overactivity	0.93	0.50	0.74	0.48	0.53	0.43

Item 2 Self-harm	0.02	0.07	0.15	0.12	0.00	0.09

Item 3 Substance abuse	0.11	0.14	0.18	0.10	0.00	0.04

Item 4 Cognitive Impairment	1.93	0.79	0.37	0.36	0.32	0.19

Item 5 Physical Impairment	0.43	0.36	0.30	0.43	0.42	0.30

Item 6 Hallucinations/delusions	0.69	1.47	0.48	0.31	0.16	0.13

Item 7 Depressed Mood	0.35	0.69	1.20	1.99	1.42	1.28

Item 8 Other mental/behaviour problems	1.00	1.18	1.60	1.43	2.11	1.82

Item 9 Relationships	1.98	1.97	1.84	1.26	0.95	1.11

Item 10 Daily living	2.07	1.60	1.03	0.86	0.42	0.49

Item 11 Living conditions	1.35	1.04	0.85	0.62	0.21	0.44

Item 12 Occupation/activities	1.17	0.99	0.64	0.52	0.26	0.33

H1-3 Behaviour total	1.06	0.71	1.07	0.70	0.53	0.56

H4-5 Impairment total	2.35	1.15	0.66	0.80	0.74	0.48

H6-8 Symptom total	2.04	3.33	3.28	3.73	3.68	3.23

H9-12 Social total	6.57	5.61	4.37	3.26	1.84	2.37

H1-12 HoNOS	12.02	10.82	9.40	8.490	6.79	6.65

To determine whether a patient's diagnostic grouping could be predicted on the basis of his/her profile of HoNOS item scores, a linear discriminant analysis was performed. Cross validated (leave-one-out) classification results show that 64% of the patients' groupings were correctly predicted. However, this was due to the high performance associated with the discriminant function, which correctly classifies 90% of schizophrenic patients (representing 58% of involved patients), whereas the remaining discriminant functions produced high cross validated error rates (ranging from 0.59 to 0.87 for other diagnostic groups), thus demonstrating the limited discriminatory power of HoNOS regarding diagnostic groups.

## Discussion

In this section we provide a critical discussion of the empirical results and offer some suggestions for improving the instrument.

The results of the presented study confirm the departure from uni-dimensionality. However, as HoNOS was developed to provide wide coverage of different aspects of mental health, the finding that the instrument is more multidimensional than unidimensional only confirms the status of its original function. Therefore, HoNOS total score need to be interpreted with caution.

Furthermore, the analysis has illustrated, under the unidimensional assumption of the Rasch model, the poor performance of the scoring process for certain items.

To this end, the appropriateness of the scoring process for selected items is a crucial factor in guaranteeing the utility and usability of the HoNOS. International evidence suggests that HoNOS ratings are less reliable when completed by clinical, rather than research staff [10]. In this perspective, the "Response process", as an additional source of Messick's construct validity [26] (i.e., the relationship between the intended construct and the thought processes of patients or raters) plays a central role. These drawbacks should be addressed in further initiatives providing the necessary training and supervision to ensure that the standardisation and quality of the rating practice is maintained.

Specifically, in the Behaviour subscale, under the assumptions of the Rasch model, Item 2 ("Self-harm") and Item 3 ("Substance abuse"), express poorly calibrated estimation difficulties regarding patients' measurements.

Moreover, item weights showed that Item 2 and Item 3 contributed modestly to the mean HoNOS, indicating that most patients scored low on these items.

One possible explanation is that psychiatric patients may exhibit symptoms of depression or behavioural problems even when their case history is not considered severe and their compensations are acceptable in other dimensions. Another possible explanation for low average scores for Item 2 and Item 3 could be the process of patient selection in the Italian Mental Health sector. Mental Hospitals with wards for long-term patients no longer exist in Italy, having been definitively closed by 1999. The service has evolved from the devolution of mental hospitals into Mental Health Departments which, for the purpose of appropriateness, accept only patients with comorbidities related to mental disorders, whereas patients with other (not psychotic) pathologies (organic disease, severe suicidal tendencies or disability problems) were treated by different services (medical settings or social assistance). Furthermore, in the Lombardy region, patients with drug and alcohol addiction, typically treated by different delegated services, were accepted by MHDs only if they had comorbidities of severe mental illness.

Furthermore, Rasch item fit statistics demonstrate a mechanism of "compensation" bias for the Item 5 ("Physical Impairment"), Item 7 ("Depressed Mood") and Item 8 ("Other mental/behavioural problems"). Special care must be taken in the process of scoring for Item 7 and Item 8 which, presenting many unexpected residuals, reduce internal consistency (Item 7 also reduces the temporal stability). On the contrary, raters' scores tend to amplify problems in Daily living (Item 10) and Relationships (Item 9). Item weights also showed that the contribution of Item 9 to the mean HoNOS exceeded that of the other items, reflecting the prevalence of interpersonal problems among patients with mental illness.

High average scores for Item 9 and Item 10 can be justified by clinical considerations. Often, negative symptoms in many behavioural situations (Item 7, Item 8), and also in chronic situations (Item 5), are typically and easily intercepted by Item 10 (Daily living), rather than the Symptomatic Problems subscale (Item 6, 7 and 8). Secondly, these items, not necessarily being related to "psychiatric dimensions", typically underlie mental disorders only when particular events (e.g. violence or aggression) occur. Third, Item 8, due to the large number of symptoms it encompasses, could be separated into different sub-items, thus allowing independent measurement of components linked to different domains. In fact, when specific information on symptoms encompassed by item 8 is absent, the psychometric properties of such item were found to be only sufficient to moderate [4,6,7,9,40,41].

The second analysed source of validity ('relations with other variables') is controversial. Item scores have an acceptable power of discrimination for patients among settings: setting groups scored the highest on those items most germane to their illness, as demonstrated in other studies [7,9]. Discriminant Analysis confirms this finding as well. On the other hand, the items provide limited discrimination power for the prediction of differences in illness groups, as represented by diagnoses.

The empirical results illustrated in the present paper are confirmed by other studies in literature. Specifically, numerous authors found evidence against uni-dimensionality [19-21,42,43]. As far as the factorial structure is concerned, our obtained component structure reflects the same configuration of [9], but greatly differs from that obtained by [4] and other authors [19-21,42,43]. In Amin's Manchester study [5], Item 8 and the social subscale were the lowest reliable item and subscale; McClelland and colleagues [9] demonstrated that Item 7, Item 8 and Item 9 reduce the discriminatory power of the scores: surprisingly, Item 8 was scored much higher than expected relative to the other items, as a consequence of the large number of symptoms this item encompasses. Salvi and colleagues [10] show that Item 5 correlates with Item10 on the factor called "social functioning", instead of with the Impairment factor.

McClelland and colleagues [9] suggest omitting some of the social problem items to improve the discriminatory power of the scores, particularly Item 11 and Item 12, which present over-discrimination relative to other HoNOS items and requiring rigorous guidelines for scoring Item 9 and Item 10.

## Conclusions

Accordingly to many studies that have suggested alternative multidimensional factor structures for HoNOS, the present paper confirms that the HoNOS does not measure a single, underlying construct of mental health status, recommending a note of caution regarding use of a summary index of the HoNOS scores, given the presence of multidimensionality. This practice could potentially lead to an erroneous assessment of patients' psychiatric mental illness. However, violation of uni-dimensionality and item misfit occurs as the complexity of the data is typically greater than that allowed for by the axiomatic Rasch model. Though these findings suggest that responses to different items are determined by different latent traits, they confirm that under this hypothesis the items should not be expected to fit a simple unidimensional (Rasch) model.

This serves to underline the point that, by assuming a unidimensional model when the latent trait is multidimensional, generally speaking, we will be unable to make sound inferences about the real structure of the data, reinforcing the importance of performing empirical exploratory analysis, rather than remaining satisfied with unsupported assumptions. Unfortunately, the Rasch model does not explore the possibility of more than one dimension (of mental illness), providing insufficient information of an exploratory nature, particularly concerning dimensionality.

Instead, whether an instrument is useful at the service level depends on more than the stability of its total score. The HoNOS is composed of 12 scales/items, each covering a completely different dimension of mental illness, thus, subscale or Item scores have utility and validity at the clinical level in identifying the different profiles of problems in groups of patients. Hence, the finding that the instrument is more multidimensional than unidimensional does not prevent the use of the HoNOS as a clinically valid outcome scale for routine use in mental health services (routine outcome assessment).

In conclusion, this study has illustrated that, despite the highlighted limitations, item scores have demonstrated sufficient reliability (item difficulties do not vary across diagnostic groups and, except Item2, care settings) and high precision in time (11 items maintain the same structure of difficulty/severity as the total score changes between occasions), indicating that HoNOS items can be utilized as valid measurement instruments in longitudinal analyses [12,13,17]. Although HoNOS data is still scarce in Italy on a large geographic scale, it is our sincere hope that the present study will prove helpful in guiding further attempts to analyse and improve the HoNOS as a standardised assessment tool, providing a useful and effective instrument for routine use by mental health practitioners.

## Competing interests

The authors declare that they have no competing interests.

## Authors' contributions

PGL undertook the statistical analysis and contributed to the drafting of the manuscript. EM wrote the Section "Background", EM and PGL wrote the section "Discussion", PGL the remaining sections. All authors have read and approved the final manuscript.
